# Multimorbidity patterns in adult day health center clients with dementia: a latent class analysis

**DOI:** 10.1186/s12877-022-03206-0

**Published:** 2022-06-22

**Authors:** Tina Sadarangani, Carla Perissinotto, Jonelle Boafo, Jie Zhong, Gary Yu

**Affiliations:** 1 New York University Rory Meyers College of Nursing, 433 First Avenue, New York, NY 10010 USA; 2 University of California San Francisco School of Medicine, Division of Geriatrics, 490 Illinois Street, San Francisco, CA 94158 USA

**Keywords:** Alzheimer's Disease, Latent Class Analysis, Adult Day Care, Multiple Chronic Conditions

## Abstract

**Background:**

Persons living with dementia (PLWD) in adult day centers (ADCs) represent a complex and vulnerable population whose well-being is at risk based on numerous factors. Greater knowledge of the interaction between dementia, chronic conditions, and social determinants of health would enable ADCs to identify and target the use of their resources to better support clients in need of in-depth intervention. The purpose of this paper is to (a) classify PLWD in ADCs according to their level of medical complexity and (b) identify the demographic, functional, and clinical characteristics of those with the highest degree of medical complexity.

**Methods:**

This was a secondary data analysis of 3052 clients with a dementia diagnosis from 53 ADCs across the state of California between 2012 and 2019. The most common diagnosis codes were organized into 28 disease categories to enable a latent class analysis (LCA). Chi-square test, analysis of variance (ANOVA), and Kruskal-Wallis tests were conducted to examine differences among latent classes with respect to clinical and functional characteristics.

**Results:**

An optimal 4-class solution was chosen to reflect chronic conditions among PLWD: high medical complexity, moderate medical complexity, low medical complexity, and no medical complexity. Those in the high medical complexity were taking an average of 12.72 (+/− 6.52) medications and attending the ADC an average of 3.98 days (+/− 1.31) per week—values that exceeded any other class. They also experienced hospitalizations more than any other group (19.0%) and met requirements for the nursing facility level of care (77.4%). In addition, the group experienced the greatest frequency of bladder (57.5%) and bowel (15.7%) incontinence.

**Conclusions:**

Our results illustrate a high degree of medical complexity among PLWD in ADCs. A majority of PLWD not only have multimorbidity but are socially disadvantaged. Our results demonstrate that a comprehensive multidisciplinary approach that involves community partners such as ADCs is critically needed that addresses functional decline, loneliness, social isolation, and multimorbidity which can negatively impact PLWD.

## Introduction

Dementia is a chronic and progressive syndrome characterized by cognitive decline that is projected to impair 13.8 million individuals in the United States by the year 2050 [1]. The burden of disease and disability is heightened among persons living with dementia (PLWD) who also experience multimorbidity. Multimorbidity is defined as the coexistence of 2 or more chronic conditions in an individual [2], and it is associated with the need for complex intervention [3]. On average, older people have been found to have 4.6 illnesses along with dementia [4].

Research has shown that dementia can complicate other chronic conditions and vice versa. For example, coexisting chronic conditions such as hypertension, diabetes, and coronary heart disease can exacerbate cognitive dysfunction and other symptoms in people with dementia. Conversely, dementia can complicate the care of these illnesses by accelerating functional decline and increasing the potential for adverse complications [5, 6]. Research has affirmed that when multimorbidity intersects with dementia, there is greater incidence of hospitalizations [7] and readmissions [8], emergency department use [9], fragmented care, and reduced quality of life [10]. Effective models of integrated care are urgently needed that help PLWD with multimorbidity preserve function and address unmet needs to reduce costly and traumatic health care utilization.

Adult day centers (ADCs) represent one potentially vital but overlooked platform for chronic disease management and care coordination for PLWD [5]. ADCs are nonresidential community-based facilities that support the health and social needs of adults through health monitoring, socialization, meals, and assistance with activities of daily living (ADLs) [11]. ADCs can follow either a social or health/medical model. Social models place a stronger emphasis on social and recreational activities, whereas health models tend to offer a more sophisticated rehabilitation-oriented program and provide management of current health problems such as dementia [12]. ADCs that follow health models benefit from having interdisciplinary staff (eg, registered nurses, social workers, nursing aides) and deliver culturally and linguistically congruent health and social care to clients [13]. Programs embedded within ADCs that emphasize integrated care, such as the Community Based Health Home (CBHH) model or Programs of All Inclusive Care for the Elderly (PACE) [14], reduce avoidable health care utilization among clients with multimorbidity [11].

Each day in the United States, ADCs provide care to nearly 200,000 PLWD, most of whom suffer from Alzheimer’s disease and Alzheimer’s disease–related dementias (AD/ADRD) [15]. PLWD in ADCs represent a complex and vulnerable population whose well-being is at risk based on numerous factors. In addition to experiencing a high prevalence of cognitive impairment (upwards of 30% of all clients [16]), ADC clients also face disproportionately high rates of multimorbidity, poverty, disability, limited English proficiency, transportation barriers, and food insecurity [5, 17, 18]. More than 69% of clients suffer from some combination of AD/ADRD, depression, or heart disease, and 30% have a diagnosis of diabetes [16]. Adding to this, nearly 72% of ADC clients live below federal poverty lines, and 58% are racial minorities [15].

Despite the critical implications for disease trajectory, treatments, and caregiving needs, the interrelationships among dementia, concomitant disease, and social determinants of health are not well understood among those who use ADCs [18]. The scarcity of large-scale data on ADC users restricts researchers’, policymakers’, and caregivers’ understanding of ADCs’ effectiveness and impact on users’ health and functional status [19]. Greater knowledge of the interaction between dementia, chronic conditions, and social determinants of health would enable ADCs to identify and target the use of their resources to better support clients in need of in-depth intervention. However, it is not enough to simply understand the magnitude of individual chronic conditions in PLWD; a more nuanced approach to understanding multimorbidity requires identifying possible *combinations* of chronic conditions that may interact to affect disease trajectory [20]. Moreover, given the inherent clinical complexity of this population, the interaction between dementia and multimorbidity is likely to be highly variable [13]. Developing an understanding of clusters of co-occurring chronic conditions in the ADC population would allow ADCs to optimize their programming and improve the standard of care for PLWD with multimorbidity. In addition, identifying characteristics of those at the highest risk of adverse health outcomes and directing resources to them can result in reduction of costly and traumatic healthcare utilization in PLWD and yield significant cost savings to healthcare systems. Hence, the purpose of this paper is to (a) classify PLWD in ADCs according to their level of medical complexity using latent class analysis (LCA) and (b) identify the demographic, functional, and clinical characteristics of those with the highest degree of medical complexity.

## Methods

### Design and data source

The deidentified data for this retrospective analysis of ADC clients with dementia were provided by TurboTAR for the years 2012-2019. TurboTAR is the leading provider of billing and management software to ADCs in the state of California, which is home to 30% of ADCs in the United States [15]. California is one of the few states with regulations that require ADCs to collect and report data on their clients on a serial basis. It is important to note that mandatory data reporting in California ADCs is *only* required for clients whose participation in adult day services is paid for by Medicaid and is not the case for those who pay privately or through other sources. Medicaid is a public health insurance program for people in the United States with limited incomes (less than 138% of the federal poverty level) [21]. Since Medicaid is the only public payor of adult day services and insures 72% of people in American ADCs [22], the majority of people being cared for in ADCs live below federal poverty levels.

Every 6 months, an eligibility and needs assessment is conducted on Medicaid beneficiaries enrolled in ADCs using the state’s Individualized Plan of Care (IPC). The IPC is a 26-page regulatory document that can be completed within TurboTAR, and it contains information on diagnoses, medications, ADLs and instrumental activities of daily living (IADLs) based on assessments by interdisciplinary team members at the ADC [23]. It also captures information on continence, nutrition, living arrangements, health care utilization, clinical risk factors, and social determinants of health. TurboTAR deidentified the data, which were then extracted and securely shared with the principal investigator.

### Study population

The study population included individuals aged 50 and over who (a) were clients in California ADCs, between 2012 and 2019, during which the IPC remained unchanged; (b) were Medicaid beneficiaries; (c) had a clinical diagnosis—reflected in ICD-9 or ICD-10 codes—of vascular dementia, Lewy body dementia, frontotemporal dementia, Alzheimer’s disease, or dementia not otherwise specified; and (d) had at least 2 consecutive IPCs completed after enrollment reflecting 12 months of data. Clients in ADCs that did not have data-sharing agreements with TurboTAR were excluded from this analysis. Using these criteria, our sample consisted of 3053 people.

### Variables

The study variables are listed in Table 1. We grouped variables as *demographics* (eg, race, primary language, living alone), *clinical information* (eg, number of medications), *functional status* (eg, ADL, medication mismanagement), and *chronic conditions* (eg, gastrointestinal disorders, cardiovascular disease). The 28 chronic condition categories reflect the most common diagnoses—approximately the top 10%—that emerged upon reviewing the dataset.

**Table 1:** Study Variables

Demographics	Clinical Information	Functional Status	Chronic Conditions
• AgeGender • Minority status • Primary Language • Race • Living Alone	• Number of medications daily • Planned days at center per week • Incontinent of bladder • Incontinent of bowel • Therapeutic or special diet • Difficulty chewing and/or swallowing	• Activities of daily living (ADL) • Emergency Department (ED) visits baseline • Hospitalizations at baseline • Meet a nursing facility level of care • Use an adaptive device • Inappropriate affect/appearance/behavior • Poor judgement • Medication mismanagement • Self-neglect • Dementia related behavioral problem • Fall risk • Isolation • Frailty	• Hypertension • Diabetes Mellitus (DM) with Complication • Diabetes Mellitus Without Complication • Arthropathies • Thyroid Disease • Nutritional Deficiencies • Cerebrovascular Diseases • Cardiovascular Disease • Cancer • Depression • Peripheral Vascular Disease • Chronic Kidney Disease • Chronic Liver Disease • Genitourinary Disease • Lipid Disorders • Osteoporosis • Non-depressive mood Disorder • Parkinson’s Disease • Chronic Obstructive Pulmonary Disease (COPD) • Obesity • Dorsopathies • Chronic Pain • Vision Disorders/Deficits • Hearing Disorders • Gastrointestinal Disorders • Asthma • Neuropathic Pain Disorders • Gait Disorders

### Statistical analysis

Data were validated and examined for outliers; no major outliers were found. Preliminary descriptive statistics were calculated to examine sample demographics and characteristics. Latent class analysis (LCA) was then used to empirically identify classes of ADC clients with dementia reporting similar patterns of multiple chronic conditions at the time of enrollment. In the LCA, which is based on structural equation modeling, groups, or classes, are formed by uncovering hidden, or latent, patterns of association between nonordinal observations in a dataset. LCA was the preferred method to assess patterns of multimorbidity in ADC clients because unlike descriptive approaches which would yield counts or frequencies of individual chronic conditions, LCA enables us to establish groupings based on co-occurrence of chronic conditions, and, as a result, identify broad co-morbidity patterns. LCA is a frequently used methodology in analyzing multimorbidity, because it not only enables the identification of clusters of chronic conditions, but also allows examination of differences between clusters in terms socio-demographics, function, and clinical characteristics.

The parameters of the LCA model included the following: (1) the creation of a total count indicator as a simple comorbidity score of all of the co-occurring chronic conditions present to reflect the cumulative exposure to multimorbidity; (2) the probability of each specific chronic condition present within each latent class; (3) the overall proportion of the population in each of the latent classes; and (4) the mean number of comorbid conditions present in each latent class. We fit the LCA model using maximum likelihood in Mplus version 7.31 [24], where the dichotomous chronic condition indicators were modeled with a binomial logit link and the overall count of different comorbid conditions present was modeled with a log Poisson link. The 28 dichotomous indicators of the presence of each chronic condition, as well as an overall count of the number of different chronic conditions, were analyzed using LCA with a varying number of classes, ranging from 1 to 7 [25–28]. We determined the optimal number of classes using the Bayesian information criterion (BIC), Akaike information criterion (AIC), bootstrap likelihood ratio test (BLRT), and the Lo, Mendell, and Rubin Likelihood Ratio Test (LMR-LRT) [29], which balances model fit and parsimony [30, 31] as well as clinical judgment. Once the optimal number of classes was determined, we computed the posterior probability that a certain individual belongs with a certain latent class using Bayes’ rule [32].Labels, reflecting the complexity associated with the average number of chronic conditions individuals in a latent class had, were applied to each group. We also determined that a chronic condition was disproportionately represented within a latent class if its frequency in that class was either 10% above the prevalence within the total sample or double the total prevalence.

#### Relationship between latent classes and individual characteristics

We then compared the demographic, functional, and clinical variables across classes that emerged within the LCA, using a chi-square test for categorical predictors and analysis of variance (ANOVA) for continuous predictors. We conducted post hoc multiple comparison tests using a Bonferroni correction, and then we completed Kruskal–Wallis tests in lieu of ANOVA for non-normal distributions of continuous predictors. These analyses were conducted within SPSS (version 25.0).

### Ethical considerations

The institutional review board at the principal investigator’s (TS) institution classified this deidentified secondary data analysis as nonhuman subjects research.

## Results

### Sample description

The study sample comprised 3053 ADC clients with a dementia-related diagnosis. The sample was disproportionately female (67.1), with a mean age of 79.57 years (+/− 9.44), taking 9.67 medications (+/− 6.59), and attending the ADC 3.59 days a week (+/− 1.72). Significant data (> 30%) were missing for race and English proficiency, which were nonmandatory collection fields. For those who had complete data, 42.2% were non-English speakers, and 32.4% identified as non-White.

### Latent class analysis

An optimal 4-class solution was chosen based on comprehensive evaluation of fit indices (BIC, AIC, entropy, LMR-LRT, and the BLRT). Group 1 (*n* = 801; 26.24%) consisted of individuals with an average of 5.67 chronic conditions in addition to their dementia diagnosis. They were deemed as having “high medical complexity.” Group 2 (*n* = 1120, 36.69%) had, on average, 3.91 conditions and was labeled “moderate medical complexity.” Group 3 (*n* = 802, 26.27%) had, on average, 2.07 chronic conditions and was labeled “low medical complexity.” Group 4 (*n* = 330, 10.81%) had none of the 28 chronic conditions and was considered to have “no medical complexity.” Tables 2 and Table 3 (at the end before References) present the baseline characteristics of the overall sample and the individuals assigned to the 4 groups.

**Table 2:** Percentages and average number of chronic conditions across 4 latent classes

Chronic Condition	Total Sample (%)	High Medical Complexity (%)	Moderate Medical Complexity (%)	Low Medical Complexity (%)	No Medical Complexity (%)
Hypertension	61.9	73.6 ^#^	84.9 ^#^	43.4	0
DM with Complications	9.1	15.7	11.0	3.3	0
DM without Complications	22.5	24.1	35.5 ^#^	12.3	0
Arthropathies	32.3	53.7 ^#^	35.0	19.1	0
Thyroid Disease	11.8	18.5	13.0	7.9	0
Nutritional Deficiencies	6.8	12.4	6.1	4.6	0
Cerebrovascular Diseases	6.8	11.6	7.3	3.9	0
Cardiovascular Diseases	18.7	30.6 ^#^	23.7	6.8	0
Cancer	3.6	4.9	3.8	3.5	0
Depression	25.6	44.4 ^#^	23.6	18.5	0
Peripheral Vascular Disease	3.1	6.4 ^#^	2.8	1.2	0
Chronic Kidney Disease	9.1	13.7	12.6	3.2	0
Chronic Liver Disease	0.9	2.0 ^#^	0.2	0.9	0
Genitourinary Disease	11.4	22.9 ^#^	7.2	9.4	0
Lipid Disorders	37.4	48.2 ^#^	59.0 ^#^	12.1	0
Osteoporosis	17.3	26.9	19.3	11.3	0
Mood Disorder	17.3	32.9 ^#^	11.6	15.3	0
Parkinson’s Disease	2.0	3.8	0.7	2.6	0
COPD	4.4	11.3 ^#^	1.9	2.3	0
Obesity	1.7	2.4	2.6	0.3	0
Dorsopathies	7.6	17.6 ^#^	4.7	4.1	0
Chronic Pain	1.4	3.5 ^#^	0.5	0.8	0
Hearing Disorders	5.0	11.6 ^#^	2.9	3.0	0
Visual Disorders/Deficits	5.9	12.2 ^#^	4.6	3.2	0
Asthma	4.7	9.3 ^#^	3.9	2.7	0
Gastrointestinal Disorders	13.6	32.3 ^#^	8.8	5.8	0
Neuropathic Pain Disorders	3.6	9.1 ^#^	2.5	0.6	0
Gait Disorders	4.8	11.5 ^#^	1.1	4.7	0
Average Number of Chronic Conditions	3.50	5.67	3.91	2.07	0
N (%)	3053 (100.0)	801 (26.24)	1120 (36.69)	802 (26.27)	330 (10.81)

*Notes*: *DM* Diabetes Mellitus, *COPD* Chronic Obstructive Pulmonary Disease

^#^indicated that the prevalence of chronic condition within the class was either 10% above the prevalence within the total sample or double the total prevalence

**Table 3:** Demographic, clinical, and functional status information across 4 latent classes

Variables	Total Sample Mean ± SD or N (%)	High Medical Complexity Mean ± SD or N (%)	Moderate Medical Complexity Mean ± SD or N (%)	Low Medical Complexity Mean ± SD or N (%)	No Medical Complexity Mean ± SD or N (%)
**Demographics**					
Age ^+^	79.57 ± 9.44	79.40 ± 9.27	80.14 ± 8.44	78.88 ± 10.58	79.74 ± 10.02
Gender ^+^					
Female	2049 (67.1)	544 (67.9)	779 (69.6)	503 (62.7)	223 (67.6)
Male	1001 (32.8)	257 (32.1)	340 (30.4)	297 (37.0)	107 (32.4)
Minority status *					
White	642 (21.0)	223 (27.8)	207 (18.5)	173 (21.6)	39 (11.8)
Minority	990 (32.4)	255 (31.8)	404 (36.1)	243 (30.3)	88 (26.7)
Primary language *					
English	602 (19.7)	154 (19.2)	179 (16.0)	205 (25.6)	64 (19.4)
Non-English	1289 (42.2)	393 (49.1)	510 (45.5)	296 (36.9)	90 (27.3)
Living alone with no family *	462 (15.2)	149 (18.6)	187 (16.7)	108 (13.5)	18 (5.7)
**Clinical information**					
Number of medications daily *	9.67 ± 6.59	12.72 ± 6.52	9.82 ± 6.49	8.07 ± 5.77	5.62 ± 5.46
Planned days at center per week *	3.59 ± 1.72	3.98 ± 1.32	3.84 ± 1.44	3.78 ± 1.58	1.31 ± 2.05
Incontinent of bladder *	1440 (47.2)	462 (57.7)	547 (48.8)	390 (48.6)	41 (12.4)
Incontinent of bowel *	415 (13.6)	126 (15.7)	152 (13.6)	116 (14.5)	21 (6.4)
Therapeutic or special diet *	1433 (46.9)	430 (53.7)	670 (59.8)	308 (38.4)	25 (7.6)
Difficulty chewing and/or swallowing *	196 (6.4)	70 (8.7)	50 (4.5)	67 (8.4)	9 (2.7)
**Functional status**					
Any emergency department visits at baseline *	473 (15.5)	142 (17.7)	198 (17.7)	116 (14.5)	17 (5.2)
Any hospitalizations at baseline	435 (14.2)	152 (19.0)	154 (13.8)	112 (14.0)	17 (5.2)
Meet nursing facility level of care *	1834 (60.4)	620 (77.4)	637 (56.9)	499 (62.5)	78 (24.6)
Use an adaptive device *	261 (8.5)	31 (3.9)	102 (9.1)	102 (12.7)	26 (7.9)
Inappropriate affect/appearance/behavior *	743 (24.3)	203 (25.3)	262 (23.4)	239 (29.8)	39 (11.8)
Poor judgement *	1701 (55.7)	488 (60.9)	642 (57.3)	504 (62.8)	67 (20.3)
Medication mismanagement *	1231 (40.3)	392 (48.9)	463 (41.3)	338 (42.1)	38 (11.5)
Self-neglect	933 (30.6)	260 (32.5)	343 (30.6)	294 (36.7)	36 (10.9)
Dementia related behavioral problems *	1344 (44.0)	394 (49.2)	475 (42.4)	412 (51.4)	63 (19.1)
Fall risk *	1924 (63.0)	598 (74.7)	762 (68.0)	497 (62.0)	67 (20.3)
Isolation *	1730 (56.7)	500 (62.4)	686 (61.2)	475 (59.2)	69 (20.9)
Frailty *	927 (30.4)	311 (38.8)	335 (29.9)	253 (31.5)	28 (8.5)
***Activity of daily living***					
Ambulation *					
Independent	338 (11.1)	71 (8.9)	126 (11.3)	120 (15.0)	21 (6.4)
Needs Supervision	1514 (49.6)	449 (56.1)	607 (54.2)	408 (50.9)	50 (15.2)
Needs Assistance	551 (18.0)	172 (21.5)	213 (19.0)	149 (18.6)	17 (5.2)
Dependent	75 (2.5)	27 (3.4)	24 (2.1)	23 (2.9)	1 (0.3)
Bathing *					
Independent	117 (3.8)	30 (3.7)	46 (4.1)	35 (4.4)	6 (1.8)
Needs Supervision	705 (23.1)	206 (25.7)	286 (25.5)	192 (23.9)	21 (6.4)
Needs Assistance	1477 (48.4)	436 (54.4)	573 (51.2)	415 (51.7)	53 (16.1)
Dependent	168 (5.5)	46 (5.7)	59 (5.3)	53 (6.6)	10 (3.0)
Dressing *					
Independent	331 (10.8)	94 (11.7)	127 (11.3)	97 (12.1)	13 (3.9)
Needs Supervision	809 (26.5)	244 (30.5)	335 (29.9)	209 (26.1)	21 (6.4)
Needs Assistance	1192 (39.0)	343 (42.8)	457 (40.8)	342 (42.6)	50 (15.2)
Dependent	137 (4.5)	36 (4.5)	48 (4.3)	47 (5.9)	6 (1.8)
Self-feeding *					
Independent	1304 (42.7)	387 (48.3)	531 (47.4)	350 (43.6)	36 (10.9)
Needs Supervision	861 (28.2)	255 (31.8)	330 (29.5)	244 (30.4)	32 (9.7)
Needs Assistance	272 (8.9)	68 (8.5)	98 (8.8)	88 (11.0)	18 (5.5)
Dependent	33 (1.1)	8 (1.0)	7 (0.6)	15 (1.9)	3 (0.9)
Toileting *					
Independent	555 (18.2)	151 (18.9)	236 (21.1)	150 (18.7)	18 (5.5)
Needs Supervision	1119 (36.7)	337 (42.1)	457 (40.8)	295 (36.8)	30 (9.1)
Needs Assistance	703 (23.0)	204 (25.5)	241 (21.5)	224 (27.9)	34 (10.3)
Dependent	94 (3.1)	26 (3.2)	33 (2.9)	28 (3.5)	7 (2.1)
Transferring *					
Independent	462 (15.1)	98 (12.2)	173 (15.4)	163 (20.3)	28 (8.5)
Needs Supervision	1281 (42.0)	380 (47.4)	537 (47.9)	330 (41.1)	34 (10.3)
Needs Assistance	686 (22.5)	224 (28.0)	244 (21.8)	190 (23.7)	28 (8.5)
Dependent	52 (1.7)	16 (2.0)	18 (1.6)	17 (2.1)	1 (0.3)

*Notes*: + *p* < 0.05, * *p* < 0.001. Minority included Black, Native Americans, Asians, Pacific Islander, Other Race, and Multiple Race; and there are 46.5% of missing data on minority status and 38.1% of missing data on primary language preference

### ADC clients with dementia and high medical complexity

#### Demographic characteristics

The average age of clients with high medical complexity was 79.40 (+/− 9.27) years, and 67.9% were female. This was largely consistent with the sample overall. For those who had no missing data on race and language, 49.1% were non-English speakers, higher than any other group, and 31.8% identified as non-White. The proportion of people in the high medical complexity group who lived alone (18.6%) was higher than in any other group.

### Functional characteristics

Relative to all other groups, ADC clients in the high medical complexity group experienced the greatest frequency of (a) difficulty chewing and/or swallowing (8.7%), (b) incontinence of the bladder (57.5%), and (c) incontinence of the bowel (15.7%). They were also the most frequently classified as needing assistance and/or being dependent with ambulation (24.9%), bathing (60.1%), and transferring (30.0%). Notably, the high medical complexity group had the lowest use rate of adaptive devices (3.9%), were more likely to be at risk for falls (74.7%) and experienced the highest degree of social isolation (62.4%). In addition, nearly half (49.2%) of individuals in the high medical complexity group experienced dementia-related behavioral problems. Notably, those in the low medical complexity group required greater assistance and were dependent with regard to self-feeding, bathing, and toileting compared to all other groups. Nearly half (48.9%) mismanaged their medications, more so than any other group. The low medical complexity group also had a slightly greater proportion group of people with poor judgment (62.8% vs. 60.9%) and inappropriate affect, appearance, or behavior (29.8% vs. 25.3%) compared to the high medical complexity.

### Clinical characteristics

ADC clients with dementia who were classified as having high medical complexity were disproportionately impacted by 18 chronic conditions. These conditions, which occurred at least 10% above the total prevalence, are denoted in Table 2 with an asterisk (*). These include hypertension, arthropathies, cardiovascular diseases, depression, peripheral vascular disease, chronic, liver disease, genitourinary disease, lipid disorders, mood disorder, COPD, dorsopathies, chronic pain, hearing disorders, visual disorders/deficits, asthma, gastrointestinal disorders, neuropathic pain disorders, and gait disorders. In the high medical complexity group, individuals were taking 12.72 (+/− 6.52) medications and attending the ADC 3.98 days (+/− 1.31) per week—values that exceeded any other class. A greater proportion of people with high medical complexity experienced hospitalizations compared to any other group (19.0%). In this group, 77.4% of PLWD met requirements for nursing facility level of care, which is defined by the California Department Aging as requiring the level of intensive care provided by a skilled nursing facility/nursing home.

## Discussion

The purpose of this paper was to (a) classify PLWD in ADCs according to their level of medical complexity and (b) identify the demographic, functional, and clinical characteristics of those with the highest degree of medical complexity. Of the 3053 PLWD in our sample, 26.24% were classified as having high medical complexity. Among other conditions, these individuals experienced disproportionately high rates of cardiovascular disease, depression, genitourinary disease, gastrointestinal disorders, and neuropathic pain in addition to cognitive impairment.

The extant literature points to patients with multimorbidity as being high utilizers of health care who are “costly” and “difficult to treat.” [33, 34] Care of older adults with multimorbidity who attend ADCs is often complicated by problems with mobility, limited English proficiency, poverty, cognitive impairment, disability and food insecurity [5, 18, 35, 36]. Our data suggest that PLWD in the centers are no exception. In ADCs, staff are potentially supporting people with multiple chronic conditions who are also cognitively impaired, functionally dependent, impoverished, frequently do not speak English, and experience polypharmacy.

In addition to having multiple chronic conditions that contribute to high medical complexity, in more than 1/4 of our sample, we also saw a high degree of functional dependence, particularly with respect to ambulation, bathing, and transferring. Those with high medical complexity also experienced the greatest frequency of gait, hearing, and visual disorders, and were at risk of falls. Simultaneously, they experienced the lowest rate of use of adaptive devices compared to any other group, suggesting a possible unmet need in this population.

Other factors that heightened the vulnerability of highly medically complex persons with dementia in ADCs are high rates of limited English proficiency (49.2%), their propensity to live alone in spite of having dementia (18.6%), and a high degree of social isolation (62.4%) relative to other groups. Another concerning finding is that, although individuals in this group were taking on average more than 12 medications, nearly half (48.9%) mismanaged their medications. The possible impact of medical complexity and social risk factors on health care utilization among ADC users with dementia is evident in our data. Nearly 1/5 had experienced a hospitalization in the prior 6 months, and approximately 1/3 met requirements for a nursing facility level of care [37].

Proper management of multimorbidity is one of the greatest health-related challenges facing patients, caregivers, health care providers, and payors [31–36], particularly among PLWD. In a survey of clinicians, clinical leaders, and executives at organizations globally that are directly involved in care delivery by the *New England Journal of Medicine*, 67% reported their organizations do not offer multidisciplinary care, and 62% reported care fragmentation as the biggest barrier to dementia care delivery [38]. There has been much emphasis on coordinating, integrating, and effectively managing the health of individuals with complex health and social needs in order to reduce avoidable health care utilization. The inherent complexity of the ADC population, as evidenced within our data, suggests it is no longer acceptable to continue to have siloed medical systems and community-based support networks. Systematic reviews of the literature call for innovative integrated patient-centered approaches that empower patients and their caregivers in a team-based format [19, 33]. Models such as PACE and CBHH show that these types of programs, which can address both health and social needs, can be effective when headquartered in the ADC.

PACE is a community-based health care program serving people who are over the age of 55 and who require nursing home–level care but prefer to receive it in their own communities [14]. Services are delivered primarily in an ADC, and they are managed by an interdisciplinary team that includes a geriatric physician as well as nurses, social workers, and therapists. A number of studies provide evidence that PACE is effective in reducing nursing home and hospital utilization and improving health status and quality of life [39, 40].

The CBHH model delivers transitional care support, patient activation, and education to advance health literacy to high-needs individuals in ADCs [5]. These services are provided through the inclusion of a registered nurse navigator (RN-N) within the interdisciplinary team at the ADC [5]. The RN-N deepens understanding of the highest risk individuals’ unique challenges and social environments by conducting health and psychosocial assessments, making home visits whenever needed, and facilitating care transitions [5].

One of the greatest barriers to expanding and scaling comprehensive ADC-based programs targeting people with complex needs is reimbursement. Despite facilitating health management and promotion for medically complex PLWD, these centers may not be adequately reimbursed for the services they are providing, which include preventive health services, cognitive and behavioral health services, and nutrition. Medicaid currently reimburses ADCs an average of $74 per day for this level of care [41, 42]. Many centers have been forced to shut down altogether in the wake of the COVID-19 pandemic [43], potentially leaving a major gap in services for high-needs PLWD. Given that ADCs serve a diverse clientele—58% of whom identify as racial/ethnic minorities—this gap in access to ADCs will likely have a disproportionate effect on communities of color and undermine health equity [16].

In the absence of unlimited funding, information and communication technology represents a scalable and economical approach to integrating ADCs into the care continuum that supports comprehensive coordination of health and social support for PLWD with multimorbidity [44, 45]. Members of the care team (eg, patients, informal caregivers, pharmacists, nurses, social workers) can communicate more seamlessly across settings with the help of shared information systems that leverage technology [46]. Electronic health records, telemonitoring systems, or mobile health applications can support bidirectional information sharing between patients and providers, as well as among individual providers, that lends itself to integrated care for PLWD [45]. However, currently, 92% of ADCs lack the resources to implement interoperable electronic health record systems [15]. Given the ubiquity of tablets and mobile phones, mobile applications that enable secure communication and information exchange may be a starting point for improving communication across the community-based care continuum. This would enable ADC staff to advocate on behalf of their clients when they are in other settings.

Our study is not without limitations. First, the sample was limited to adult day *health* centers. ADCs can follow either a social or health/medical model. The selection of medical model ADCs may have biased our sample toward more frail and complex older adults. Furthermore, our sample was limited to the state of California. Although California is home to 1/3 of adult day programs nationally, and our dataset is one of the largest available on ADC users with dementia, future research should look at trends and variability in ADC users at a national level. Also, our analysis does not include dementia severity assessments which could help explain higher levels of functional dependence in persons with low medical complexity relative to those with high or moderate complexity. In future analyses we plan to examine the interplay between dementia severity and medical complexity. Finally, assessments of participants within the IPC were based on the clinical judgment of social workers and nurses as opposed to validated instruments. This may have led to more inaccuracies or variabilities in assessments than a more standardized approach would have lent itself to.

## Conclusion

Health care, including functional and social support for people with multiple health conditions, is not straightforward, and the addition of cognitive impairment adds a further layer of complexity. For future service development to best support the growing number of people with these conditions, the first stage is to describe this population and to understand the interaction between dementia and other common comorbidities. Such an interaction is not simple and is highly variable. Our results should also serve as a signal to primary care providers (PCPs) and health systems about the degree of complexity of clients seen at ADCs. At the very least, if a patient is receiving services at an ADC, this should be a loud signal for their PCP that they need help, are at risk of complications, and are at high risk of nursing home placement. This also means that the patients seen do not just experience multimorbidity but are socially disadvantaged. Our results demonstrate that a comprehensive multidisciplinary approach that involves community partners such as ADCs is critically needed that addresses functional decline, loneliness, social isolation, and multimorbidity which can negatively impact PLWD.

## Data Availability

The data that support the findings of this study are available from Tina Sadarangani, PhD but restrictions apply to the availability of these data, which were used under license for the current study, and so are not publicly available. Data are however available from the authors upon reasonable request and with permission of Tina Sadarangani, PhD.

## Abbreviations

ADC: Adult Day Care
ADLs: Activities of Daily Living
AIC: Akaike Information Criterion
ANOVA: Analysis of Variance
BIC: Bayesian Information Criterion
BLRT: Bootstrap Likelihood Ratio Test
CBHH: Community Based Health Home
COPD: Chronic Obstructive Pulmonary Disease
COVID: Coronavirus Disease
DM: Diabetes Mellitus
ED: Emergency Department
IPC: Individualized Plan of Care
IADLs: Instrumental Activities of Daily Living
LCA: Latent Class Analysis
LMR-LRT: Lo, Mendell, and Rubin Likelihood Ratio Test
PACE: Programs of All-Inclusive Care for the Elderly
PCP: Primary Care Provider
PLWD: Persons Living with Dementia
RN-N: Registered Nurse Navigator

## References

[1] Hebert LE, Weuve J, Scherr PA, Evans DA (2013). Alzheimer disease in the United States (2010–2050) estimated using the 2010 census. Neurology.

[2] World Health Organization. Multimorbidity. World Health Organization; 2016. https://apps.who.int/iris/handle/10665/252275. Accessed Nov 12, 2021.

[3] Uijen AA, van de Lisdonk EH (2008). Multimorbidity in primary care: prevalence and trend over the last 20 years. Eur J Gen Pract.

[4] Guthrie B, Payne K, Alderson P, McMurdo MET, Mercer SW (2012). Adapting clinical guidelines to take account of multimorbidity. BMJ.

[5] Sadarangani T, Missaelides L, Eilertsen E, Jaganathan H, Wu B (2019). A mixed-methods evaluation of a nurse-led community-based health home for ethnically diverse older adults with multimorbidity in the adult day health setting. Policy Polit Nurs Pract.

[6] Maslow K (2004). Dementia and serious coexisting medical conditions: a double whammy. Nurs Clin North Am.

[7] Phelan EA, Borson S, Grothaus L, Balch S, Larson EB (2012). Association of incident Dementia with hospitalizations. JAMA.

[8] Hirschman K, Shaid E, McCauley K, Pauly M, Naylor M (2015). Continuity of care: the transitional care model. OJIN Online J Issues Nurs.

[9] Voss S, Black S, Brandling J (2017). Home or hospital for people with dementia and one or more other multimorbidities: what is the potential to reduce avoidable emergency admissions? The HOMEWARD project protocol. BMJ Open.

[10] Naylor MD, Feldman PH, Keating S (2009). Translating research into practice: transitional care for older adults. J Eval Clin Pract.

[11] Oliver RE, Foster M (2013). Adult day care: an Important Long-Term Care Alternative & Potential Cost Saver. Mo Med.

[12] Leitsch SA, Zarit SH, Townsend A, Greene R (2001). Medical and social adult day service programs: a comparison of characteristics, dementia clients, and their family caregivers. Res Aging.

[13] Welsh TJ (2019). Multimorbidity in people living with dementia. Case Rep Womens Health.

[14] National PACE Association (2021). National PACE association.

[15] National Center for Health Statistics, ed. Long-term care providers and services users in the United States, 2015-2016. Hyattsville: U.S. Department of Health and Human Services, Centers for Disease Control and Prevention, National Center for Health Statistics; 2019.

[16] Caffrey C, Lendon JP (2019). Service provision, hospitalizations, and chronic conditions in adult day Services centers: findings from the 2016 National Study of long-term care providers. Natl Health Stat Rep.

[17] Aging In Place. Adult Day Care. AgingInPlace.org. 2021. https://aginginplace.org/adult-day-care/. Accessed Jun 30, 2021.

[18] Sadarangani TR, Murali KP (2018). Service use, participation, experiences, and outcomes among older adult immigrants in American adult day service centers: an integrative review of the literature. Res Gerontol Nurs.

[19] Sadarangani T, Anderson K, Vora P, Missaelides L, Zagorski W. A national survey of data currently being collected by adult day service centers across the United States. J Appl Gerontol. 2021:07334648211013974. 10.1177/07334648211013974 Published online May 14. 10.1177/07334648211013974 33985380

[20] Quiñones AR, Kaye J, Allore HG, Botoseneanu A, Thielke SM (2020). An agenda for addressing multimorbidity and racial and ethnic disparities in Alzheimer’s disease and related dementia. Am J Alzheimers Dis Dementias®.

[21] Services D of HC (2022). Medi-Cal Eligibility.

[22] California Department of Health Care (2021). Medi-Cal.

[23] California Department of Health Care. Individual plan of care. 2021. https://www.aging.ca.gov/download.ashx?lE0rcNUV0zZUdBVNwHKfkg%3D%3D. Accessed 4 Nov 2021.

[24] Muthén LK, Muthén BO (2017). Mplus user’s guide.

[25] Murtaugh C, Peng T, Totten A, Costello B, Moore S, Aykan H (2009). Complexity in geriatric home healthcare. J Healthc Qual.

[26] Yu G, Goldsamt LA, Clatts MC, Giang LM (2016). Sexual initiation and complex recent Polydrug use patterns among male sex Workers in Vietnam: a preliminary epidemiological trajectory. Arch Sex Behav.

[27] Yu G, Wall MM, Chiasson MA, Hirshfield S (2015). Complex drug use patterns and associated HIV transmission risk behaviors in an internet sample of U.S. men who have sex with men. Arch Sex Behav.

[28] Cheung YK, Yu G, Wall MM, Sacco RL, Elkind MSV, Willey JZ (2015). Patterns of leisure-time physical activity using multivariate finite mixture modeling and cardiovascular risk factors in the northern Manhattan study. Ann Epidemiol.

[29] Nylund KL, Asparouhov T, Muthén BO (2007). Deciding on the number of classes in latent class analysis and growth mixture modeling: a Monte Carlo simulation study. Struct Equ Model Multidiscip J.

[30] McLachlan GJ, Chang SU (2004). Mixture modelling for cluster analysis. Stat Methods Med Res.

[31] Fraley C (1998). How many clusters? Which clustering method? Answers via model-based cluster analysis. Comput J.

[32] Rindskopf D, Rindskopf W (1986). The value of latent class analysis in medical diagnosis. Stat Med.

[33] Smith SM, Soubhi H, Fortin M, Hudon C, O’Dowd T (2012). Managing patients with multimorbidity: systematic review of interventions in primary care and community settings. BMJ.

[34] Wallace E, Salisbury C, Guthrie B, Lewis C, Fahey T, Smith SM (2015). Managing patients with multimorbidity in primary care. BMJ.

[35] Sadarangani TR, Johnson JJ, Chong SK, Brody A, Trinh -Shevrin Chau. (2020). Using the social ecological model to identify drivers of nutrition risk in adult day settings serving east Asian older adults. Res Gerontol Nurs.

[36] Sadarangani TR, Missaelides L, Yu G, Trinh-Shevrin C, Brody A (2019). Racial disparities in nutritional risk among community-dwelling older adults in adult day health care. J Nutr Gerontol Geriatr.

[37] California Department of Aging. Level of care criteria: California Code of Regulations (CCR). https://www.aging.ca.gov/download.ashx?lE0rcNUV0zatdWya3ruHJA%3D%3D. Accessed 21 June 2022.

[38] Sawyer RJ. The growing challenge of Dementia care. NEJM Catal. 2(9). 10.1056/CAT.21.0285.

[39] Chatterji P, Burstein NR, Kidder D, White A (1998). The Impact of PACE on Participant Outcomes FINAL.

[40] Nadash P (2004). Two models of managed long-term care: comparing PACE with a Medicaid-only plan. The Gerontologist.

[41] Barlow J, Wright C, Sheasby J, Turner A, Hainsworth J (2002). Self-management approaches for people with chronic conditions: a review. Patient Educ Couns.

[42] Boyd CM, Darer J, Boult C, Fried LP, Boult L, Wu AW (2005). Clinical practice guidelines and quality of care for older patients with multiple comorbid diseases implications for pay for performance. JAMA.

[43] Sadarangani T, Zhong J, Vora P, Missaelides L (2021). “Advocating every single day” so as not to be forgotten: factors supporting resiliency in adult day service centers amidst COVID-19-related closures. J Gerontol Soc Work.

[44] Steele Gray C, Mercer S, Palen T, McKinstry B, Hendry A (2016). eHealth advances in support of people with complex care needs: case examples from Canada, Scotland and the US. Healthc Q.

[45] Suter E, Arndt J, Arthur N, Parboosingh J, Taylor E, Deutschlander S (2009). Role understanding and effective communication as core competencies for collaborative practice. J Interprof Care.

[46] Wenzl M (2019). New ways of delivering care for better outcomes.

